# Supplementary material for: Early-stage lung cancer is driven by a transitional cell state dependent on a KRAS-ITGA3-SRC axis
Source: EMBO J. 2024 May 16;43(14):3. doi: 10.1038/s44318-024-00113-5 (PMC11251082; doi:10.1038/s44318-024-00113-5)
Supplement: Supplementary file 1 — Dataset EV1 [file 44318_2024_113_MOESM1_ESM.zip › Figure_Legends_for_Dataset_EV1.docx]

**Dataset EV1: Differentially expressed genes (DEGs) in the scRNA-seq organoid dataset, based on genotype and time point.** List of DEGs from AT2 cells that were subset from the organoid scRNA-seq dataset. The cells were grouped based on genotype and time point. Gene name, log fold change, and statistical significance are provided, and DEGs were determined using the in-built scanpy.tl.rank_genes_groups() function and parameters in Scanpy (Wolf, Angerer, and Theis 2018).

**References**

Wolf, F. Alexander, Philipp Angerer, and Fabian J. Theis. 2018. “SCANPY: Large-Scale Single-Cell Gene Expression Data Analysis.” *Genome Biology* 19 (1). https://doi.org/10.1186/s13059-017-1382-0.
