# Supplementary material for: Early-stage lung cancer is driven by a transitional cell state dependent on a KRAS-ITGA3-SRC axis
Source: EMBO J. 2024 May 16;43(14):3. doi: 10.1038/s44318-024-00113-5 (PMC11251082; doi:10.1038/s44318-024-00113-5)
Supplement: Supplementary file 2 — Dataset EV2 [file 44318_2024_113_MOESM2_ESM.zip › Figure_Legends_for_Dataset_EV2.docx]

**Dataset EV2: DEGs in the scRNA-seq *Rosa26^YFP^* organoid dataset, based on Leiden community.** Lists of DEGs from *Rosa26^YFP^* AT2 cells subset from the organoid scRNA-seq dataset. The cells were grouped based on Leiden community. DEGs from our AT1 and AT2 intermediate state analysis are also provided. Gene name, log fold change, and statistical significance are provided, and DEGs were determined using the in-built scanpy.tl.rank_genes_groups() function and parameters in Scanpy (Wolf, Angerer, and Theis 2018).

**References**

Wolf, F. Alexander, Philipp Angerer, and Fabian J. Theis. 2018. “SCANPY: Large-Scale Single-Cell Gene Expression Data Analysis.” *Genome Biology* 19 (1). https://doi.org/10.1186/s13059-017-1382-0.
