# Supplementary material for: Early-stage lung cancer is driven by a transitional cell state dependent on a KRAS-ITGA3-SRC axis
Source: EMBO J. 2024 May 16;43(14):3. doi: 10.1038/s44318-024-00113-5 (PMC11251082; doi:10.1038/s44318-024-00113-5)
Supplement: Supplementary file 3 — Dataset EV3 [file 44318_2024_113_MOESM3_ESM.zip › Figure_Legends_for_Dataset_EV3.docx]

**Dataset EV3: Differentially expressed genes in scRNA-seq *Kras^G12D^ p53^flox^* *Rosa26^YFP^* organoid dataset, based on Leiden cluster.** List of DEGs from *Kras^G12D^ p53^flox^* *Rosa26^YFP^* AT2 cells subset from the organoid scRNA-seq dataset. The cells were grouped based on Leiden community. Gene name, log fold change, and statistical significance are provided, and DEGs were determined using the in-built scanpy.tl.rank_genes_groups() function and parameters in Scanpy (Wolf, Angerer, and Theis 2018).

**References**

Wolf, F. Alexander, Philipp Angerer, and Fabian J. Theis. 2018. “SCANPY: Large-Scale Single-Cell Gene Expression Data Analysis.” *Genome Biology* 19 (1). https://doi.org/10.1186/s13059-017-1382-0.
