# Supplementary material for: Early-stage lung cancer is driven by a transitional cell state dependent on a KRAS-ITGA3-SRC axis
Source: EMBO J. 2024 May 16;43(14):3. doi: 10.1038/s44318-024-00113-5 (PMC11251082; doi:10.1038/s44318-024-00113-5)
Supplement: Supplementary file 4 — Dataset EV4 [file 44318_2024_113_MOESM4_ESM.zip › Figure_Legends_for_Dataset_EV4.docx]

**Dataset EV4: DEGs for LUAD initiation analysis from mouse, organoid, and stage IA patient samples.** DEGs were identified in the AT2 cell component of each model systems scRNA-seq dataset (organoid, mouse, and stage IA patient). Cells were grouped based on Leiden community. Gene name, log fold change, and statistical significance are provided, and DEGs were determined using the in-built scanpy.tl.rank_genes_groups() function and parameters in Scanpy (Wolf, Angerer, and Theis 2018).

**References**

Wolf, F. Alexander, Philipp Angerer, and Fabian J. Theis. 2018. “SCANPY: Large-Scale Single-Cell Gene Expression Data Analysis.” *Genome Biology* 19 (1). https://doi.org/10.1186/s13059-017-1382-0.
