# Supplementary material for: Early-stage lung cancer is driven by a transitional cell state dependent on a KRAS-ITGA3-SRC axis
Source: EMBO J. 2024 May 16;43(14):3. doi: 10.1038/s44318-024-00113-5 (PMC11251082; doi:10.1038/s44318-024-00113-5)
Supplement: Supplementary file 5 — Dataset EV5 [file 44318_2024_113_MOESM5_ESM.zip › Figure_Legends_for_Dataset_EV5.docx]

**Dataset EV5: GeneWalk Input and Results.** DEGs in the Day 4 *Kras^G12D^ p53^flox^* *Rosa26^YFP^* AT2 cluster (C5) relative to Day 4 *Rosa26^YFP^* AT2 cluster (C8). C5 DEGs are expressed in MGI annotation as this is the gene nomenclature used as input for GeneWalk. The output of our GeneWalk analysis is also included.

**References**

Wolf, F. Alexander, Philipp Angerer, and Fabian J. Theis. 2018. “SCANPY: Large-Scale Single-Cell Gene Expression Data Analysis.” *Genome Biology* 19 (1). https://doi.org/10.1186/s13059-017-1382-0.
