# Supplementary material for: Early-stage lung cancer is driven by a transitional cell state dependent on a KRAS-ITGA3-SRC axis
Source: EMBO J. 2024 May 16;43(14):3. doi: 10.1038/s44318-024-00113-5 (PMC11251082; doi:10.1038/s44318-024-00113-5)
Supplement: Supplementary file 10 — Expanded View Figures [file 44318_2024_113_MOESM10_ESM.pdf]

Expanded View Figures

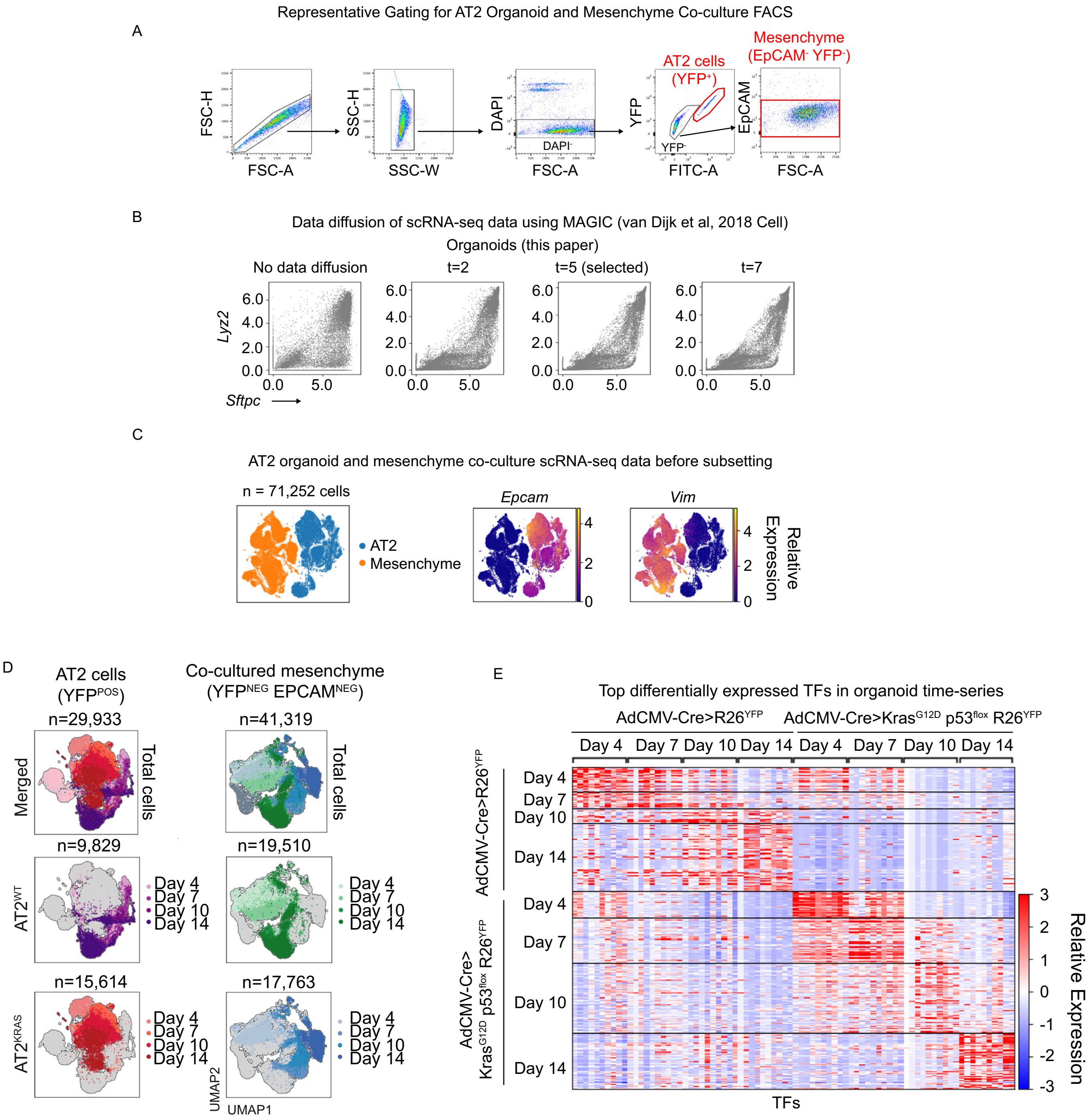

Figure EV1. Generation and initial characterization of the AT2-mesenchyme organoid co-culture scRNA-seq dataset.

(**A**) Representative FACS plot for AT2-mesenchyme co-culture time course for scRNA-seq analysis. (**B**) Correlation between *Sftpc* and *Lyz2* expression in individual organoid co-culture cells, after different levels of data diffusion (*t*) (van Dijk et al, 2018). (**C**) UMAP representation of filtered single cells from organoid co-cultures before subsetting, their corresponding population of origin, and *Epcam* or *Vim* expression. (**D**) UMAP representations of filtered single cells from organoid co-cultures and their corresponding population of origin, genotype, and time point. (**E**) Heatmap of the top 10 differentially expressed transcription factors in AT2 cells based on genotype and time point.

© The Author(s)

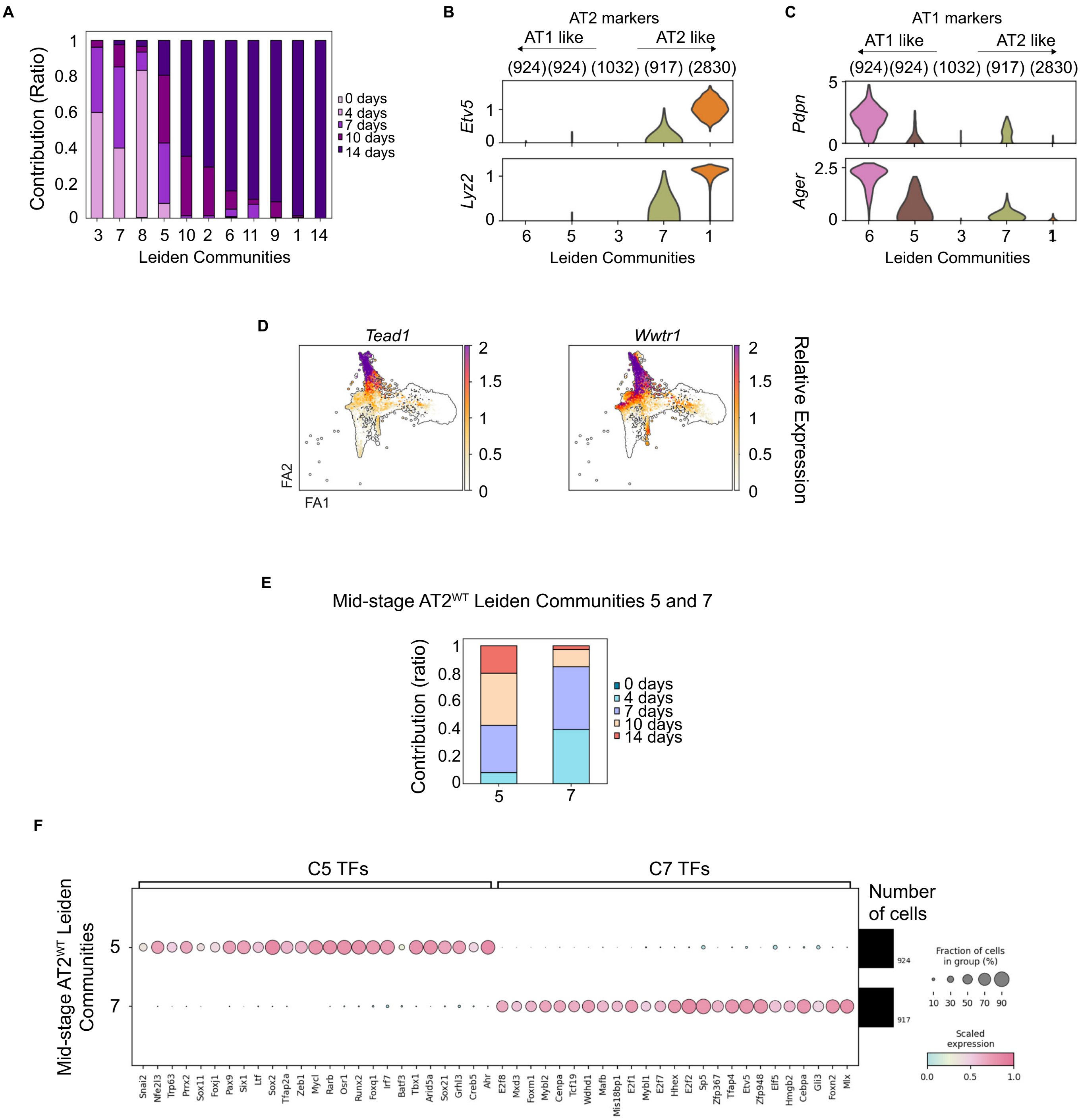

Figure EV2. Additional characterization of the AT2^{WT} organoid scRNA-seq dataset.

(**A**) Bar plot representing time point contributions in each Leiden community in the AT2^{WT} organoid scRNA-seq data, represented as a ratio. Leiden communities consisting primarily of Day 0 AT2 cells were excluded from the barplot. (**B**) Relative expression of AT2 genes *Etv5* and *Lyz2* per community using a violin plot (*y*-axis, Leiden community; *x*-axis, relative expression). (*n*) denotes the number of cells per cluster. (**C**) Relative expression of AT1 genes *Pdpn* and *Ager* per community using a violin plot (*y*-axis, Leiden community; *x*-axis, relative expression). (**D**) FA2 representations of filtered single cells subset from AT2^{WT} organoid data and the relative expression of either *Tead1* or *Wwtr1*. (**E**) Bar plot representing time point contributions to AT2^{WT} organoid intermediates C5 (AT1 fate) and C7 (AT2 fate). (**F**) Relative expression of the top 25 DE TFs in C5 and C7 AT2^{WT} intermediate states using a dot plot (*x*-axis; DE genes, *y*-axis; Leiden community).

 © The Author(s)

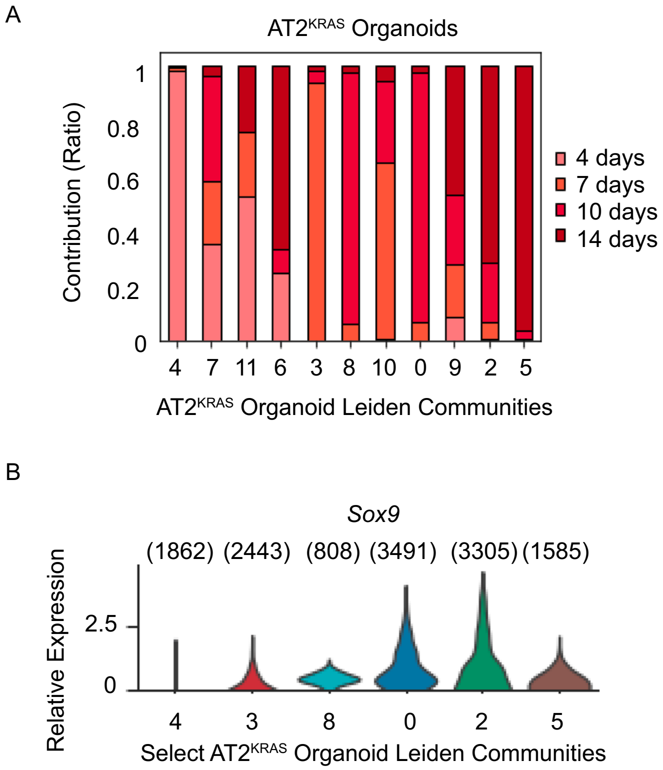

Figure EV3. Additional characterization of the AT2^{KRAS} organoid scRNA-seq dataset.

(**A**) Barplot representing time point contributions in each Leiden community in the AT2^{KRAS} organoid scRNA-seq data, represented as a ratio. (**B**) Relative *Sox9* expression in select early-, mid-, and late-stage communities using a violin plot (*y*-axis, relative *Sox9* expression; *x*-axis, Leiden community). (*n*) denotes the number of cells per cluster.

© The Author(s)

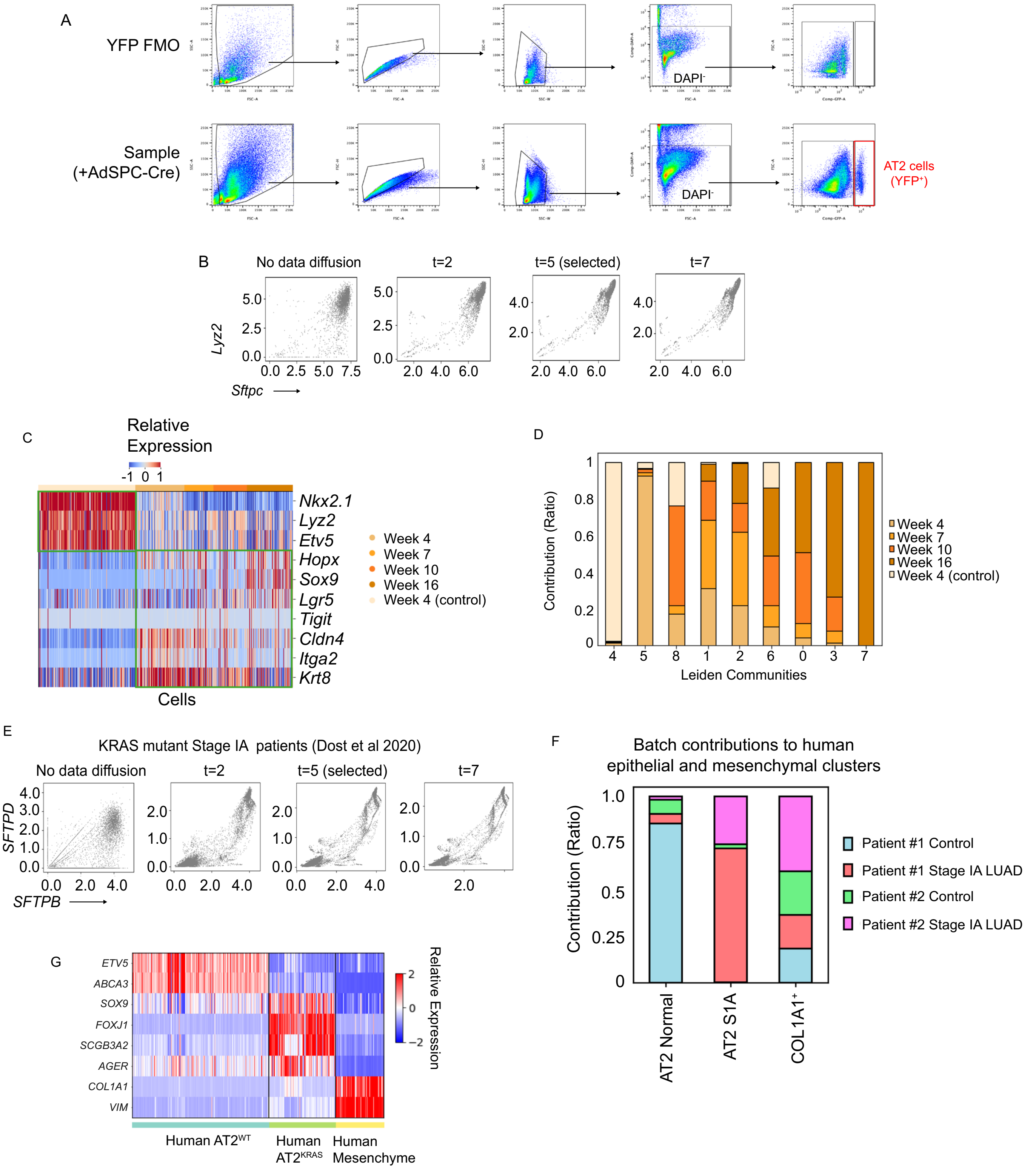

Figure EV4. Additional characterization of the in vivo and stage IA human scRNA-seq datasets.

(**A**) Representative FACS plot for in vivo time course for scRNA-seq experiment. (**B**) Correlation between *Sftpc* and *Lyz2* expression in vivo, in individual AT2 cells after different levels of data diffusion (t) (van Dijk et al, 2018). (**C**) Heatmap of gene expression in the in vivo time course data relevant to AT2, AT1, development, stem cell, and injury response gene expression signatures. (**D**) Bar plot representing time point contributions in each Leiden community in the in vivo scRNA-seq data, represented as a ratio. (**E**) Correlation between *SFTPD* and *SFTPB* expression in individual stage IA human cells after different levels of data diffusion (*t*) (van Dijk et al, 2018). (**F**) Bar plot representing patient and sample type batch contributions for each Leiden community in the human scRNA-seq data, represented as a ratio. (**G**) Heatmap of gene expression in human data relevant to different lung lineages and development.

 © The Author(s)

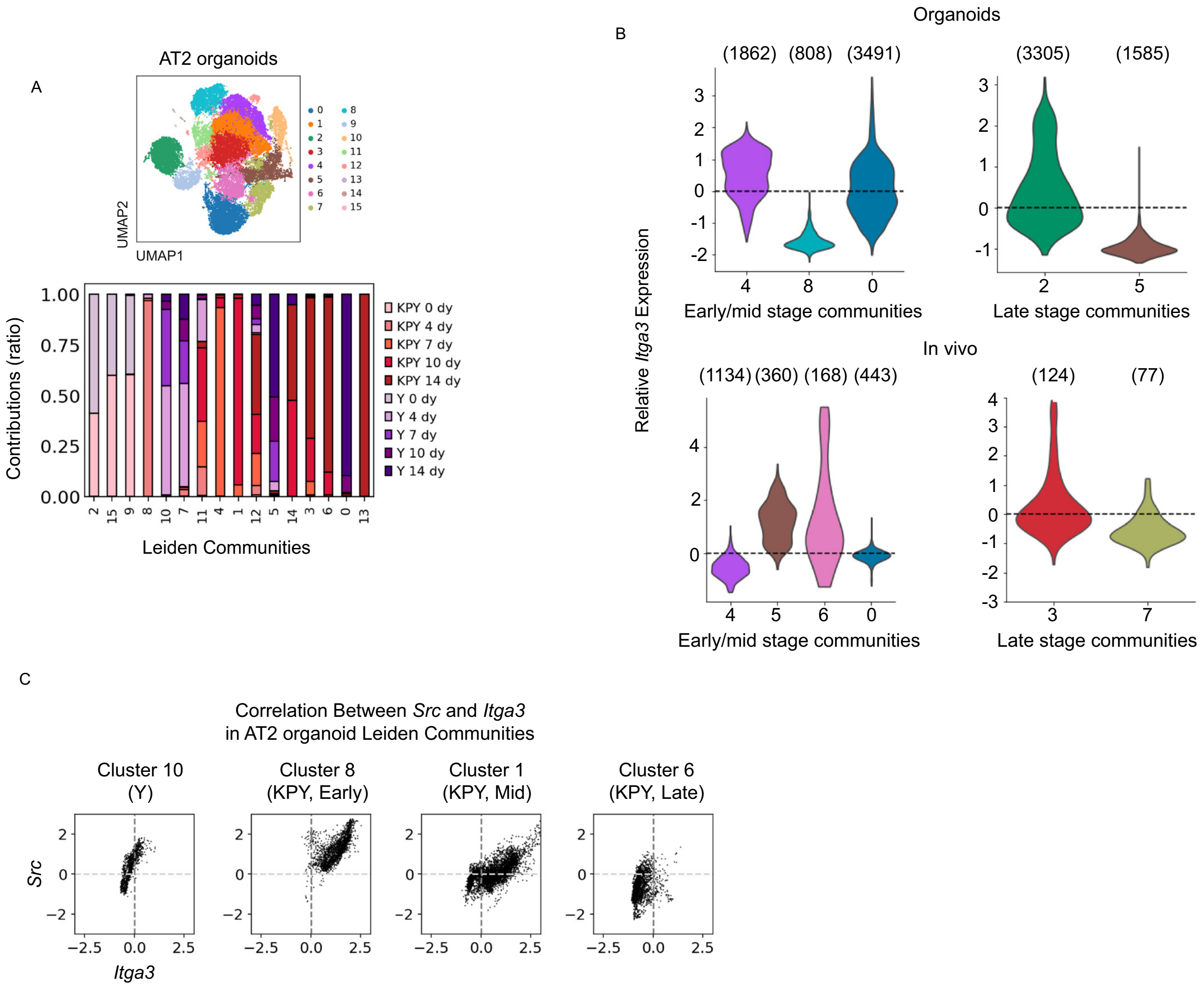

Figure EV5. Additional analysis related to Fig. 4.

(**A**) UMAP representation of Leiden communities in the combined AT2 organoid data. Bar plot representing time point contributions for each Leiden community, represented as a ratio. (**B**) Relative *Itga3* expression in AT2^{KRAS} organoids (top) and in vivo (bottom) Leiden communities using a violin plot, subset into early-/mid- and late-stage time points (*y*-axis, relative *Itga3* expression; *x*-axis, Leiden community). (**C**) Correlation between *Src* and *Itga3* relative expression at various time points in AT2 organoids, represented as a scatterplot. Each point represents a single cell.

© The Author(s)
